# Synergistic Effect of Garcinol and Curcumin on Antiproliferative and Apoptotic Activity in Pancreatic Cancer Cells

**DOI:** 10.1155/2012/709739

**Published:** 2012-05-23

**Authors:** Mansi A. Parasramka, Smiti Vaid Gupta

**Affiliations:** Department of Nutrition and Food Science, College of Liberal Arts and Sciences, Wayne State University, 3009 Science Hall, Detroit, MI 48201, USA

## Abstract

Pancreatic cancer (PaCa) is a major health concern due to its aggressiveness and early metastasis. Current treatments for PaCa are limited by development of resistance against therapy. As an alternative strategy, we assessed the combinatorial effect of dietary compounds, garcinol and curcumin, on human PaCa cells (BxPC-3 and Panc-1). A significant (*P* < 0.05) dose-dependent reduction in cell viability and increase in apoptosis were observed in both cell lines as compared to untreated controls. A combination index (CI) value < 1, for a two-way comparison of curcumin and garcinol, suggests synergism. The potency (*Dm*) of the combination of garcinol and curcumin was 2 to 10 fold that of the individual agents. This indicates that curcumin and garcinol in combination exhibit a high level of synergism, with enhanced bioactivity, thereby reducing the required effective dose required for each individually. This combinatorial strategy may hold promise in future development of therapies against PaCa.

## 1. Introduction

Cancer is a major health concern across the globe today with pancreatic cancer (PaCa) being the fifth major cause of deaths due to cancer in the United States. Development and progression of this chronic disease involves deregulation and activation of multiple signaling pathways at different stages of carcinogenesis. This complexity associated with the disease causes limitations in designing high-efficacy therapeutic strategies. Although immense work has been done in the prevention and treatment of PaCa, the results are not satisfactory and need improvement. For example, gemcitabine is a standard cytotoxic chemotherapeutic agent used for treatment in PaCa. However, this drug provides limited survival advantage along with several side effects and development of chemoresistance [1, 2]. Therefore, exploring new approaches for better maintenance of this disease is of great interest.

Epidemiological studies have consistently shown that consumption of a healthy diet including fruits, vegetables, and whole grains is strongly associated with reduced risk of cancer and other diseases [3]. Over the years, several natural bioactive compounds or phytochemicals have been isolated, identified, and their potential anticarcinogenic properties have been evaluated. However, efficacy of interactions amongst various dietary components needs further analysis. There is a growing body of evidence that chemotherapeutic combination strategies would be more efficient in reducing drug toxicity, inhibiting tumor development and progression than with either agent alone. Research suggests that increased consumption of curcumin isolated from *Curcuma longa *or Turmeric, contributes to lower risk of cancer in Asian (Indian) populations as compared to industrialized western nations [4, 5]. Also recently, the anticancer properties of garcinol, a bioactive compound derived from the rind of the fruit, *Garcinia indica,* Kokum or Mangosteen has been revealed. The anticarcinogenic properties of this compound in several cancers such as breast, oral, and colon cancer have been tested. Very recently, we and others have showed that garcinol reduces pancreatic cancer cell viability and increases apoptosis via downregulation of nuclear factor NF-*κ*B [6, 7]. Asian (Indian) populations consume turmeric and kokum as a spice in their diet regularly. It is possible that the lower incidence of pancreatic cancer in India (according to 2008 statistics, 2 cases per 100,000 persons per year) as compared to the western nations (in 2008, an estimated 37,680 new cases of pancreatic cancer (18,770 in men and 18,910 in women) were diagnosed in the United States) [3, 8] might be attributed in part from interactions between the bioactive components in diet including that of turmeric and kokum.

When a combination of two or more compounds exhibit a more potent therapeutic effect than that of individual compounds at equal concentrations, the effect is described to be a synergistic one. In this study, we tested the hypothesis that the bioactive compounds garcinol from *Garcinia indica* and curcumin derived from *Curcuma longa* will work in synergism and inhibit the growth of PaCa cells. Therefore, garcinol, curcumin, and their combination were tested in PaCa cell lines BxPC-3 and Panc-1, carrying wildtype and mutated *K-ras,* respectively. Our results indicate that the combination of garcinol and curcumin significantly inhibited cell viability (*P* < 0.05) and caused induction of apoptosis via upregulation of caspase-3 and -9 activity (*P* < 0.05) in both cell lines. The combination proved to be synergistic and/or additive. These data suggest a potential for the combination therapy for improvement of efficacy in PaCa treatment and hence warrants further investigation.

## 2. Material and Methods

### 2.1. Cell Culture

 Human pancreatic carcinoma cell lines BxPC-3 and Panc-1 were obtained from American Type Culture Collection (Manassas, VA, USA). The cell lines were maintained in continuous exponential growth by twice a week passage in RPMI-1640 medium (Cellgro Manassas, VA; BxPC-3 cells) and Dulbecco modified Eagle's medium (*DM*EM, Cellgro Manassas, VA; Panc-1 cells), respectively and supplemented with 10% fetal bovine serum, 100 units/mL penicillin, and 10 mg/mL streptomycin in a humidified incubator containing 5% CO_2_ in air at 37°C. Each cell line was split regularly before attaining 70–80% confluence.

### 2.2. Reagents

Garcinol (≥95% (TLC), Biomol International, USA) and curcumin (≥94% (curcuminoid content), ≥80% (Curcumin) Sigma Aldrich, USA) were dissolved in *DM*SO to make 20 mM stock solution.

### 2.3. Cell Viability

 Cells were seeded at a density of 3 × 10^3^ cells/well in 96-well microtiter culture plates. After overnight incubation, the medium was removed and replaced with fresh medium containing different concentrations of garcinol (0–40 *μ*M) diluted from a 20 mM stock and/or curcumin (0–50 *μ*M). After 72 hrs, MTS (3-(4,5-dimethylthiazol-2-yl)-5-(3-carboxymethoxyphenyl)-2-(4-sulfophenyl)-2H-tetrazolium, inner salt) solution was added to each well and incubated further for one hour. Color development was measured spectrophotometrically at 595 nm on a plate reader (BIO-TEK Instruments) and quantified as per the manufacturer's protocol (Promega, USA). Cell viability has been expressed as a percentage, for each treatment group relative to control in the absence of garcinol or curcumin.

### 2.4. Quantification of Apoptosis

The cell apoptosis ELISA Detection Kit (Roche, Palo Alto, CA, USA) was used to detect apoptosis according to the manufacturer's protocol. Briefly, after treatment of BxPC-3 and Panc-1 cells with garcinol or curcumin for 48 hrs, the cytoplasmic histone/DNA fragments from cells were extracted and bound to immobilized antihistone antibody. Subsequently, a peroxidase-conjugated anti-DNA antibody was used for the detection of immobilized histone/DNA fragments. After addition of the peroxidase substrate, the absorbance by the samples was determined at 405 nm with an ULTRA Multifunctional Microplate Reader (BIO-TEK Instruments).

### 2.5. Morphological Changes

 Morphological changes characteristic of apoptosis were determined by DAPI (4′, 6-diamidino-2-phenylindole) staining as per manufacturer's protocol (Invitrogen, USA). Briefly, 5 × 10^3^ cells were seeded into 6-well plates containing 1-2 mL medium. After 24–36 hrs, garcinol and/or curcumin was added and incubated for another 48 hrs. Cells were harvested by trypsinization, washed with PBS, and subsequently incubated for 30 minutes with DAPI at room temperature in dark for 30 minutes. Prior to microscopic analysis, the cells were stained with Prolong Gold Antifade reagent and visualized under Fluorescence Microscope (Nikon Eclipse, 80i) with an excitation maximum at 358 nm and an emission maximum at 461 nm.

### 2.6. Caspase Activity

 Caspase-3 and -9 activities were measured in whole-cell lysates prepared from garcinol and/or curcumin-treated samples using a commercially available assay kit (R&D Assay System, Minneapolis, MN, USA) according to the manufacturer's instructions.

### 2.7. Analysis of Cytotoxic Synergy

Cells were plated as described above and allowed to attach overnight. The culture medium was replaced with fresh medium containing curcumin and garcinol individually or in combination in different ratios of 1 : 2.5, 1 : 5, and 1 : 10 for 72 hrs, and the effect on cell growth was examined by the MTS assay method as described above and then analyzed using the Calcusyn (Biosoft) software program, which utilizes the T.C. Chou method of determining synergy and antagonism.

The combination index was determined at a 25%, 50%, and 75% toxicity level for each cell line and at each drug ratio. The median-effect equation and combination index (CI) analysis was used to calculate the interaction between treatment modalities. This analysis determines if the effect of the combination is antagonistic, additive, or synergistic. A CI value of one indicates that the effect of one drug is additive to the second, a CI value of greater than one indicates antagonism between the two agents, and a CI value of less than one indicates synergism between the agents. The equation used to calculate CI is as follows: (*D*)1/(*Dx*)1 + (*D*)2/(*Dx*)2 + *α*(*D*)1(*D*)2/(*Dx*)1(*Dx*)2, where (*Dx*)1 and (*Dx*)2 are the doses for *x*% inhibition by drug 1 and drug 2 alone [9]. These values are obtained from the median-effect equation. (*D*)1 and (*D*)2 are the doses in combination that inhibit cell growth by *x*%. A more detailed description of degrees of synergism and antagonism is adapted from Chou and Hayball (CalcuSyn, Windows software for dose effect analysis. Cambridge: Biosoft, 1996).

## 3. Results and Discussion

### 3.1. Cell Viability

**Figure 1:**
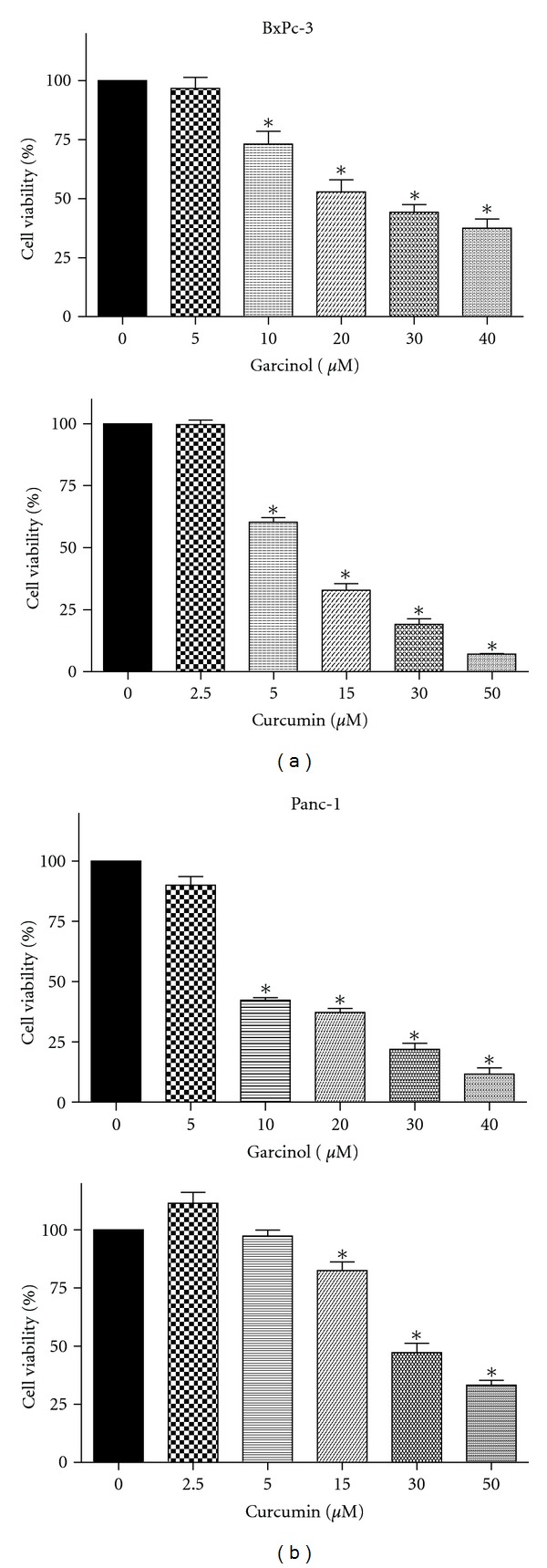
Percentage of metabolically viable cells was reduced in a dose-responsive manner on 48 hr treatment with garcinol (upper panel) or curcumin (lower panel) in both PaCa cell lines. (a) BxPC-3 and (b) Panc-1 as analyzed using MTS assay. *P < 0.05 relative to control.

BxPC-3 and Panc-1 cells were tested for their effects on cell viability under the influence of curcumin or garcinol. Both cell lines are p53 mutated with differences in their *K-ras* mutation status. Panc-1 is a poorly differentiated PaCa cell line with a mutated *K-ras,* whereas BxPC-3 is a moderately differentiated PaCa cell line with a wild type *K-ras*. We observed that both curcumin and garcinol significantly (*P* < 0.05) reduced cell viability in both cell lines in a dose-dependent manner. However, garcinol exhibited a more potent effect (IC50 = ~7 **μ**M) in Panc-1 cells which was comparable to the efficacy of curcumin (IC50 = ~10 *μ*M) in BxPC-3 cells as seen in Figure 1. Cells with mutant oncogenes such as *K-ras* continue to grow even when they are not receiving any growth signals. Both the agents reduced cell viability but to varying extents.

Garcinol and curcumin hold structural resemblance, but our results suggest that their therapeutic mechanistic targets might be different. Overall, this indicates that garcinol might play an important role in targeting the *K-ras* pathway. In BxPC-3 cells (Figure 1(a)) IC50 for garcinol treatment (upper panel) was observed at approximately 15 *μ*M as compared to 10 *μ*M curcumin treatment for 48 hours. In addition, in Panc-1 cells (Figure 1(b)) IC50 values for garcinol (upper panel) and curcumin treatment were 7 *μ*M and 25 *μ*M, respectively. We and others have consistently reported no toxicity at treatment dose of either curcumin [10] or garcinol [6, 7] on normal cells. Hence, these two agents in combination could be a useful strategy for the treatment of this aggressive disease.

### 3.2. Apoptotic Induction

**Figure 2:**
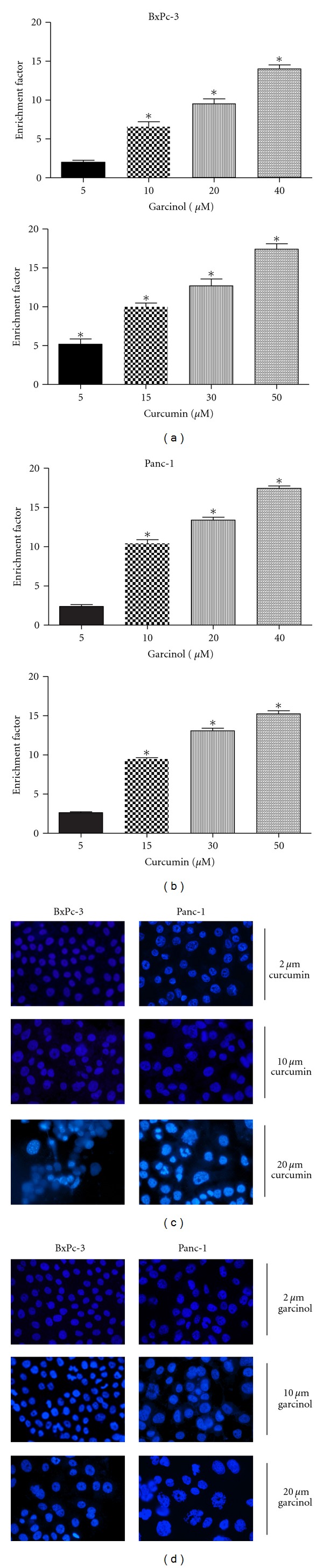
Garcinol-(upper panel) or curcumin-(lower panel) treated cytosolic extracts were used to evaluate induction of apoptosis in PaCa cells. (a) BxPC-3 and (b) Panc-1 using ELISA-Histone DNA Enrichment Assay. Results demonstrate a significant dose-dependent increase in apoptotic cells in individual treatment with either agent for 48 hours. Enrichment factor was measured using subtraction of background signal. *P < 0.05 relative to control. Apoptotic morphological changes such as abnormal nuclear morphology, reduction in cell number with apoptotic body formation, and cell shrinkage were observed in a dose responsive manner on 48 hour treatment with (c) curcumin or (d) garcinol. BxPC-3 and Panc-1 cells were fixed with DAPI stain and visualized using fluorescence microscopy with an excitation maximum at 358 nm and emission maximum at 461 nm.

 In order to confirm whether the reduction in cell viability was in part due to apoptotic induction, we performed the ELISA assays and saw that both garcinol (upper panel) and curcumin (lower panel) induced apoptosis in a dose-dependent manner in both PaCa cell lines, BxPC-3 (Figure 2(a)) and Panc-1 (Figure 2(b)). Normal cells become malignant when cellular tumor suppressor genes are rendered nonfunctional through mutations. Both Panc-1 and BxPC-3 exhibit a p53 mutation. p53 is a key tumor suppressor protein that limits cell proliferation by inducing cell cycle arrest and apoptosis in response to cellular stress. Hence, the role of p53-independent pathways such as retinoblastoma protein (Rb) or p73*α* in induction of apoptosis needs further investigation. We determined morphological changes associated with apoptosis, such as formation of apoptotic bodies and reduction in cell number using the DAPI stain as in Figures 2(c) and 2(d) in both PaCa cell lines upon treatment.

**Figure 3:**
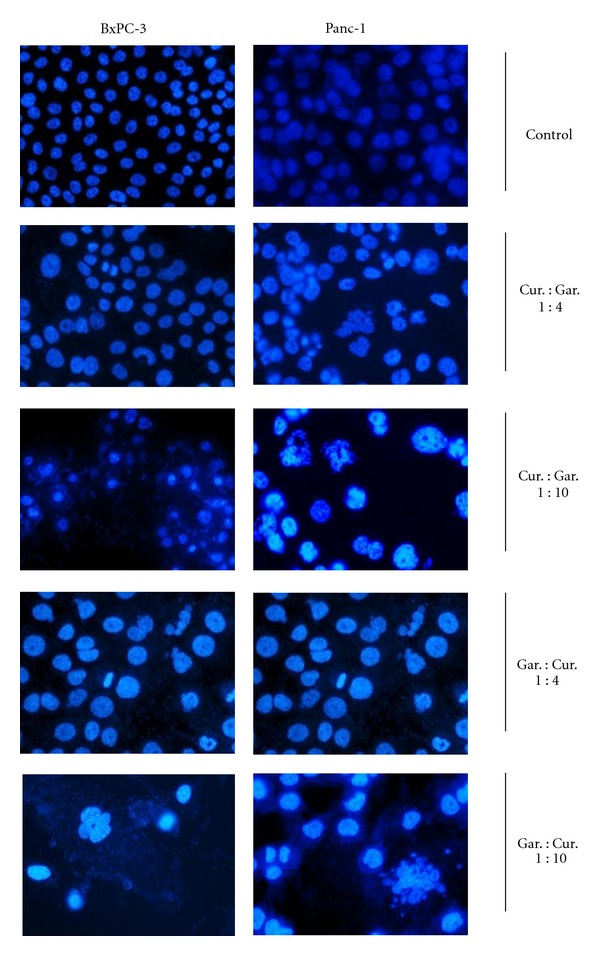
Apoptotic morphological changes such as abnormal nuclear morphology, reduction in cell number with apoptotic body formation, and cell shrinkage induced by combination treatment with curcumin and garcinol in different ratios for 48 hours were observed using DAPI stain in both PaCa cell lines: BxPC-3 (left panel) and Panc-1 (right panel). (1 : 4 ratio is 2.5 *μ*M : 10 *μ*M concentration and 1 : 10 ratio is 2 *μ*M : 20 *μ*M respective concentrations).

Both BxPC-3 (Figure 3 left panel) and Panc-1 (Figure 3 right panel) were subjected to different concentrations of curcumin and garcinol individually, and reduction in cell number along with formation of apoptotic bodies was observed as a dose-dependent effect. Figure 3 depicts the structural changes when curcumin and garcinol were given in combination in different ratios (1 : 4 ratio is 2.5 *μ*M : 10 *μ*M concentration and 1 : 10 ratio is 2 *μ*M : 20 *μ*M respective concentrations). Combination treatment had a more pronounced effect on induction of apoptosis than their respective individual treatments.

### 3.3. Caspase Activity

**Figure 4:**
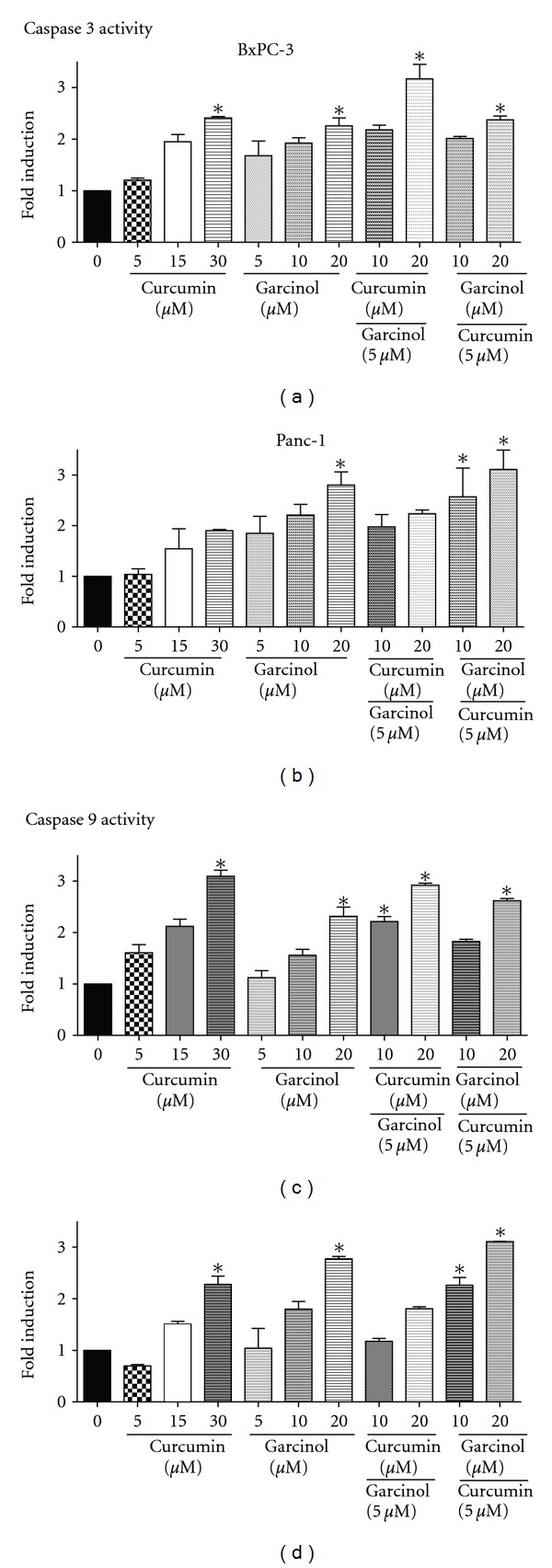
Garcinol and curcumin significantly increased Caspase-3 (a and b) and Caspase-9 (c and d) activity by ~2 to 3 folds in both PaCa cell lines: BxPC-3 (left panel) and Panc-1 (right panel) relative to untreated control after 48 hour treatment. Caspase activity was measured in garcinol- and/or curcumin-treated whole cell extracts using colorimetric assay. *P < 0.05 relative to control.

 Caspases are a family of cysteine proteases that play a very important role in apoptosis. Caspase-9 is an initiator caspase that causes the cleavage of procaspase to active form, and Caspase-3 is an executioner caspase that cleaves other protein substrates triggering the process of apoptosis. We measured caspase-3 and 9 activities using a colorimetric assay and observed a significant 2 to 3 fold induction of active caspase-3 (*P* < 0.05) (Figures 4(a) and 4(b)) and caspase-9 (*P* < 0.05) (Figures 4(c) and 4(d)) in both Panc-1 and BxPC-3 cells upon treatment with garcinol and curcumin. Similar or higher induction was observed in combination treatment with lower doses in different ratios. Caspases are usually associated with the activity of tumor suppressor genes such as p53, p73*α*, or Rb. Since both the cell lines exhibit p53 mutation, activation of caspases-3 and -9 might be due to the involvement of another tumor suppressor gene, which needs to be confirmed by further analysis.

### 3.4. Cytotoxic Synergy

**Figure 5:**
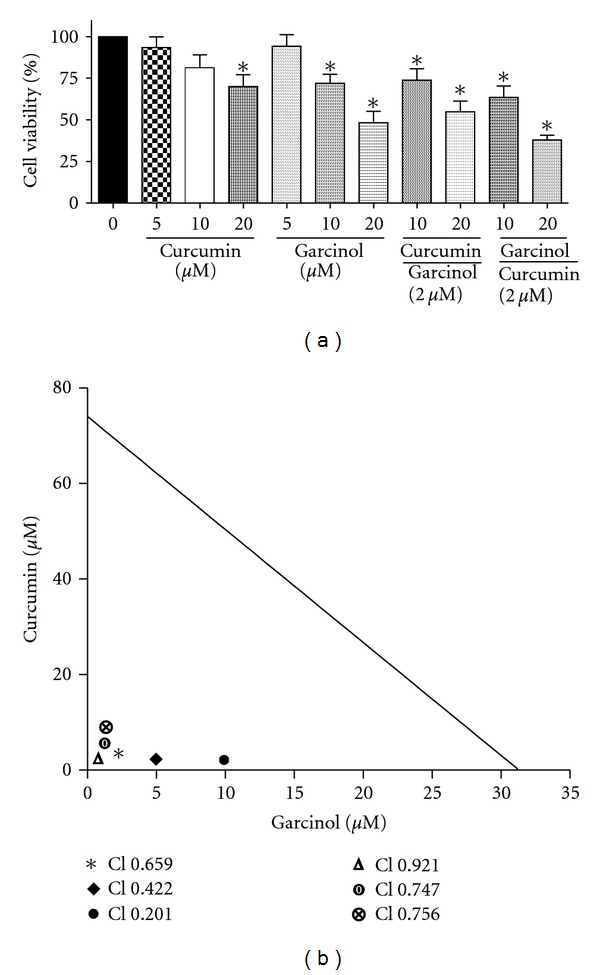
(a) combination effect of curcumin and garcinol on Panc-1 cell viability was determined using MTS assay. Combinatorial treatment significantly reduced cell viability more effectively than monotherapy on 48 hr treatment. *P < 0.05 relative to control. (b) isobologram analysis to evaluate the extent of synergism on combining curcumin and garcinol for therapeutic effect. Combination Index (CI) values at ED50 (effective dose at which 50% cells are nonviable) depicting the synergistic efficacy of garcinol and curcumin on PaCa cell line.

 In order to determine the extent of synergism between these two agents, we tested the combination effect of garcinol and curcumin in different ratios. Cell viability reduced significantly (*P* < 0.05) upon treatment in Panc-1 cells, and the effects were more pronounced in the combinatorial approach as compared to individual doses (Figure 5(a)). Using the Isobologram analysis method, we determined the combination index values (CI) for different ratios of treatment in Panc-1 cells.

Figure 5(b) shows the different CI values obtained upon treatment. CI = 1 indicates additive effect, <1 indicates synergistic effect, and >1 suggests antagonistic effect. The CI values for ED50 (effective dose for 50% inhibition) when curcumin and garcinol were administered in the ratios of 1 : 10, 1 : 5, and 1 : 2.5 were 0.201, 0.422, and 0.659, respectively. Three combinations were tested for each ratio. *Dm* can be defined as the median-effect dose or concentration signifying the potency of the treatment. It is usually depicted in correlation with ED50 values. We observed that when Panc-1 cells were treated with only curcumin and garcinol, the *Dm* values were 26 *μ*M and 17.65 *μ*M, respectively. However, when curcumin and garcinol were administered in the ratios of 1 : 10, 1 : 5 and 1 : 2.5, the *Dm* values were 4.57, 8.57, and 10.79 *μ*M, respectively. Based on the CI ED50 values and the *Dm* values, it can be concluded that there was 2 to 5 fold lower concentration requirement in combination treatment than individual treatments in order to show similar effect.

Similarly when the ratio of garcinol and curcumin was reversed, and the cells were treated with 1 : 10, 1 : 5, and 1 : 2.5 of garcinol : curcumin, the CI values at ED50 were 0.756, 0.747, and 0.921, respectively. The *Dm* values recorded for the same ratios were 1.25, 2.32, and 5.11 *μ*M, respectively. *Dm* is an important parameter which helps in establishing dose reduction leading to lowered toxicity in the host, thus retaining overall therapeutic efficacy.

Another parameter describing the sigmoidicity of the dose effect curve is the *m* value. An *m* = 1, >1, and <1 correlates with a hyperbolic, sigmoidal, and negative sigmoidal shape, respectively. We observed *m* > 1 value in all our samples except when curcumin : garcinol were in the ratio of 1 : 10 suggesting sigmoidicity of the curve. Also, *r* value is the linear correlation coefficient of the median effect plot. An *r* value equal or close to 1 indicates perfect conformity of the data. We report an *r* value close to 1 (0.97–1.00) in all our samples.

Collectively, the above results suggest that dietary interactions can be a beneficial option for the control of PaCa. This disease is a major health problem because of its increasing incidence worldwide. Given the limited therapeutic options and current unmatched clinical needs for the treatment of the patient, there is an urgent need for the development of novel agents that can influence the survival rates and quality of life for the patients. Relatively few studies have reported that the additive and synergistic effects of phytochemicals in fruits and vegetables are responsible for their potent anticancer activities. One school of thought is that this synergistic or additive effect of various bioactive compounds could be due to targeting of multiple signaling pathways. Our data clearly exhibited that garcinol in combination with curcumin had potent synergistic effect on cell viability and apoptosis.

Dietary modification is a practical approach where we can combine nontoxic phytochemicals from fruits and vegetables, and this approach may also enhance the chemotherapeutic efficacy of malignant cells with minimal toxicity to normal cells. This study demonstrates a synergistic effect between curcumin and garcinol in pancreatic cancer cells. Our findings have important implications for combination of different dietary agents for cancer therapy. However, further studies are needed to elucidate the underlying mechanisms of combinatorial approach in pancreatic cancer for enhancing efficacy and simultaneously lowering cytotoxicity to normal cells.
