# Erratum to “Synergistic Effect of Garcinol and Curcumin on Antiproliferative and Apoptotic Activity in Pancreatic Cancer Cells”

**DOI:** 10.1155/2019/7469284

**Published:** 2019-07-15

**Authors:** Mansi A. Parasramka, Smiti Vaid Gupta

**Affiliations:** Department of Nutrition and Food Science, College of Liberal Arts and Sciences, Wayne State University, 3009 Science Hall, Detroit, MI 48201, USA

In the article titled “Synergistic Effect of Garcinol and Curcumin on Antiproliferative and Apoptotic Activity in Pancreatic Cancer Cells” [1], the panel in Figure 3 showing BxPC-3 with garcinol:curcumin in a 1:4 ratio was duplicated as the panel showing Panc-1 with garcinol:curcumin in a 1:4 ratio, due to an error during the production process. The corrected Figure is shown below:

## Figures and Tables

**Figure 3:**
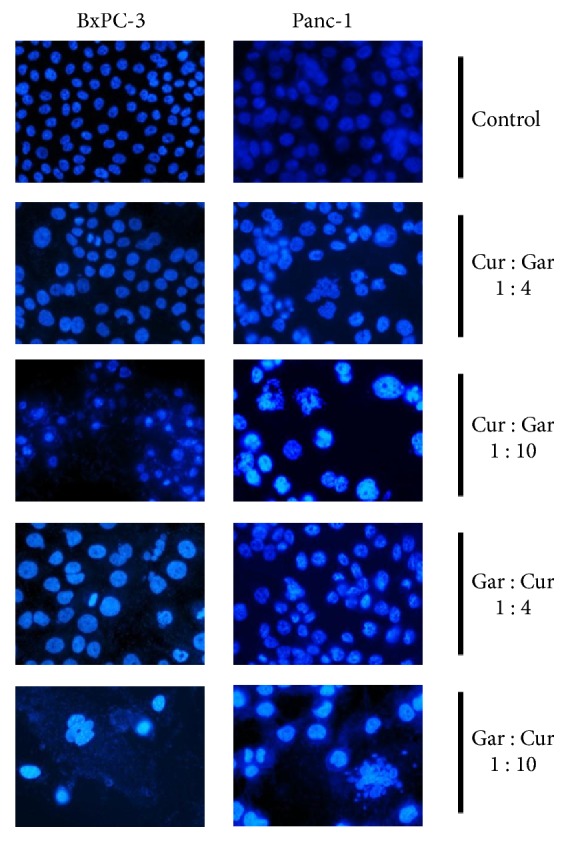
Apoptotic morphological changes such as abnormal nuclear morphology, reduction in cell number with apoptotic body formation, and cell shrinkage induced by combination treatment with curcumin and garcinol in different ratios for 48 hours were observed using DAPI stain in both PaCa cell lines: BxPC-3 (left panel) and Panc-1 (right panel) (1 : 4 ratio is 2.5 *μ*M : 10 *μ*M concentration and 1 : 10 ratio is 2 *μ*M : 20 *μ*M respective concentrations).
